# Errors in Transactions T. S. M. A.

**Published:** 1885-10

**Authors:** W. J. Burt

**Affiliations:** Secretary


### Society Notes.

### ERRORS IN THE TRANSACTIONS OF TEXAS STATE MEDICAL ASSOCIATION.

*Letter from W. J. Burt, M. D., Secretary.*

For Daniel's Texas Medical Journal.

To the Editor:

ALLOW me to call attention to two serious errors that have crept into the Transactions of 1884 and 1885:

1. At Belton, Dr. L. J. Russell introduced a resolution creating a section on "Dermatology and Medical Botany." The nominating committee appointed a chairman and secretary for two sections—one on "Dermatology" and one on "Medical Botany." This was really without authority, by misunderstanding the intention and wording of the resolution.

2. At the session at Houston, in April. 1884, Dr. F. E. Daniel, of Austin, Texas, was appointed *chairman* of the section on Dermatology by the nominating committee, but by some mistake of the secretary, the name of Dr. L. J. Russell was placed in the minutes and in the Transactions of 1885 as the chairman.

I write not as explanatory so much as to correct a mistake I made myself.

About sixty members of the association are suspended and their names dropped from the rolls of 1885 for being *over three years* behind with their *dues*. (See Transactions 1884, page 225.) These members can be *reinstated* and receive the *Transactions* of 1885 by settling the amounts they severally owe the association, either with the secretary or the treasurer.

